# Vice Explanations for Conspiracism, Fundamentalism, and Extremism

**DOI:** 10.1007/s13164-023-00685-x

**Published:** 2023-05-30

**Authors:** Rik Peels

**Affiliations:** Philosophy Department, Faculty of Religion & Theology, Vrije Universiteit Amsterdam, De Boelelaan 1105, 1081 HV Amsterdam, The Netherlands

## Abstract

In the literature on conspiracism, fundamentalism, and extremism, we find so-called vice explanations for the extreme behavior and extreme beliefs that they involve. These are explanations in terms of people’s character traits, like arrogance, vengefulness, closed-mindedness, and dogmatism. However, such vice explanations face the so-called situationist challenge, which argues based on various experiments that either there are no vices or that they are not robust. Behavior and belief, so is the idea, are much better explained by appeal to numerous situational factors, like one’s mood or how orderly one’s environment is. This paper explores the situationist challenge to vice explanations for conspiracism, fundamentalism, and extremism in more detail by assessing the empirical evidence, analyzing the argumentation based on it, and drawing conclusions for where this leaves vice explanations. The main conclusion is that vice explanations for such extreme behavior and extreme beliefs need to be fine-tuned on various points, but that there is no reason to think that they have been discredited by empirical evidence. Moreover, the situationist challenge shows that sensitivity is needed for distinguishing when vice explanations for conspiracism, fundamentalism, and extremism are appropriate, when appeal to situational factors is more fitting, and when the two can be combined.

## Introduction

Conspiracy theorizing, fundamentalism, and extremism are partly constituted by *actions* that are in some sense *extreme*: Suicide bombing, preventing women from receiving secondary education, punishing homosexuality, storming a capitol building to prevent an alleged election steal, and so on. In conspiracism, fundamentalism, and extremism, we also find a wide variety of extreme *beliefs*: That the rollout of faster 5G internet is causing or accelerating the spread of Covid-19. That Hilary Clinton is connected to a child sex ring being run out of a pizzeria in Washington DC. That the Bible is literally and infallibly true and the only source of moral knowledge. That women are the property of their husbands. That it is justified to kill those who apostatized. And so on. These beliefs are extreme in various ways: positionally they are often to the extreme right or to the extreme left, they are often based on insufficient or no evidence, they can be morally wrong, and at times they lead to harmful action. Conspiracy theorizing, fundamentalism, and extremism also come with various *affective* states, like grievances (anger, fear, perceived injustice) and *conative* states, like the desire for purity or the longing for radical redemption.^1^

Exactly how these extreme beliefs relate to extreme actions is something that I will not explore here. It is well-known that the correlation between cognitive and behavioral radicalization, for instance, is rather low (Wolfowicz et al. 2021), which is sometimes called the ‘problem of the few’: only a few out of those who have cognitively radicalized actually behaviorally radicalize. We should note though that this point holds primarily for *violent* actions, whereas we already saw that many fundamentalist, conspiracist, and extremist actions are not violent, yet harmful in other ways. It may not be likely that someone who believes violence against the West is justified for its past of colonial oppression will actually employ violence, but it is not that unlikely that someone who believes that the Bible is literally, historically, and infallibly true will actually treat the Bible in such a way, or that a conspiracy theorizer who believes we should not trust the government will actually not trust the government.

There are, of course, crucial differences between conspiracism, fundamentalism, and extremism. How exactly each of these phenomena is to be conceptualized and exactly how they relate to one another is a challenging issue that I will address in more detail elsewhere.^2^ Let me present the gist of it here. I take conspiracy theory belief to be belief (or trust, or acceptance, or some such positive propositional attitude) in a theory which posits a conspiracy as a salient cause of an event, that is, in which one distinguishes patterns of secret and hostile agency by a coalition (Van Prooijen 2018). For large-scale conspiracy theorizing, such as David Icke’s or Alex Jones’ theories, we can add that they are overarching and prima facie highly implausible. Extremism can be defined as the belief and corresponding action and affection that one’s in-group can never be successful or healthy unless it engages in hostile action towards an out-group (Berger 2018). We see it in neo-Nazism, the IRA, Salafi Jihadism, and other movements that are violent or use other extreme methods. Fundamentalism is, paradoxically, a particularly modern response to modern developments: it rejects such things as modern liberal ethics, evolutionary theory and cosmology, is modern in that it seeks certainty, sometimes formulated in ‘fundamentals’ or by adopting a literalist infallibilist reading of holy scriptures and by using modern media to spread the message, and it embraces a narrative of the world in terms of a perfect, paradisical state, a fall, and our duty to return to the original state, as well as some kind of cosmic dualism in which a battle rages between good and evil with nothing in between. It often comes with othering or even hostility. (Peels 2022; Almond et al. 2003) Conspiracism, fundamentalism, and extremism, then, display complex relationships: they can overlap but are distinct: it is possible to be a conspiracy theorizer without being a fundamentalist or extremist, one can be a fundamentalist without conspiracy theorizing and without being an extremist, etc. Much fundamentalism and extremism come with conspiracy theorizing, such as that about a Jewish elite in right-wing extremism, but we have also witnessed conspiracy theorizing turning extremist, such as at the January 6^th^ 2021 insurrection at the American Capitol.

Despite these differences between conspiracism, fundamentalism, and extremism, it can be helpful to consider these extreme phenomena together, partly because in the literature we find so-called *vice explanations* for all of them and the challenge we will identify in this paper for such vice explanations is common to all of them. As we shall see in more detail below, these explanations appeal to moral vices like aggressiveness and vengefulness, as well as cognitive vices like closed-mindedness, dogmatism, and gullibility.

There is something intuitive and common sensical about such vice explanations. For instance, it is prima facie plausible that a conspiracy theorist may believe that Covid-19 is a hoax because she is *unduly skeptical* towards any information that comes from the government, that a Christian fundamentalist may reject evolutionary theory because she is *dogmatic* and *leaves no room for doubt*, and that an ISIS fighter may believe out of *wishful thinking* that they will expel the Iraqi army from Mosul. Of course, not all fundamentalists and extremists hold extreme beliefs. Some may join a fundamentalist or extremist movement merely because they admire a particular leader or because the group provides them with a certain identity, even if they have their doubts about various views of the movement. But surely many of them, their leaders in particular, embrace such extreme beliefs. I hasten to add that their beliefs should be explained by much more than merely an appeal to vices: affections, such as various grievances, and conative states (their desires and purposes) also play a role, perhaps even macro-factors like economic, social, and political circumstances. Yet, most would agree that such vices do play *at least some important explanatory role*.^3^

However, intuition and alleged common sense can be misleading. And, in fact, vice explanations *have* come under attack. Based on different kinds of empirical inquiry, it has been argued that our common-sense picture of vices is misguided. Whether we act rightly or wrongly much more depends on the situation one is in than on any stable moral and cognitive character traits. This position has understandably been dubbed ‘situationism’. The purpose of this paper is, therefore, to assess the empirical evidence and particularly the arguments based on such evidence: does it follow that vice explanations for conspiracism, fundamentalism, and extremism are untenable?

There are at least two reasons why it matters whether people’s character traits can or cannot figure in explanations. First, if there are no stable and robust character traits, then much of the literature on fundamentalism, extremism, and conspiracism is *radically misguided*. As I shall show in this paper, vice explanations figure widely when it comes to explaining extreme behavior and extreme beliefs. Second, if situationism is true and vice explanations fail, the entire idea that people are often *responsible* and can be held *accountable* for their beliefs and the actions they perform partly on the basis of those beliefs is in jeopardy. After all, what would explain their actions and beliefs is not anything that has to do with *their* character or personality, but only with situational factors, sometimes entirely random ones, that are beyond their control.

The paper is structured as follows. After a few preliminary comments on vice and vice explanations (§2), I sketch various vice explanations for extreme behavior and extreme belief as we find them in the literature (§3). Subsequently, I summarize the situationist critique of vice explanations (§4). After that, I assess the evidence that situationists adduce and their reasoning based on that evidence, arguing that situationism does not defeat vice explanations (§5). I also show, though, that there is much to be learned from situationism and point out how vice explanations need to be fine-tuned to do justice to what we have learned from situationism (§6). Finally, I reply to various objections that one might level against my defense of vice explanations for conspiracism, fundamentalism, and extremism (§7).

## Preliminaries on Vice Explanations

Before we have a more detailed look at the literature that provides vice explanations for conspiracism, fundamentalism, and extremism, let us try to get a firmer grip on the notion of vice explanations.

First, there are both *moral* and *cognitive* vices (for various expositions, see Battaly 2010). Among the *moral* vices are: arrogance, avarice, callousness, cowardice, cruelty, cupidity, deceitfulness, dishonesty, disloyalty, envy, gluttony, greed, impatience, injustice, laziness, promiscuity, selfishness, and vengefulness. However, there is increasingly attention for so-called *cognitive* vices^4^: character traits that stand in the way of acquiring knowledge and understanding.^5^ Among the *cognitive* vices are: closed-mindedness or narrow-mindedness, dogmatism, epistemic blindness, folly, gullibility, intellectual dishonesty, obtuseness, self-deception, superficiality of thought, superstition, epistemic self-indulgence, intellectual pride, negligence, cowardice, conformity, idleness, carelessness, rigidity, prejudice, insensitivity to detail, willful naïveté, and wishful thinking.^6^ Cognitive vices, then, are habits or styles of thought, inquiry, and reflection, in particular ways of seeking and assessing evidence. Often, such vices come in pairs, as there are all sorts of relations between them. What makes these *cognitive* vices is that they are detrimental primarily *not* because they would harm individuals or nature, but because they render it more likely that one maintains false beliefs and other kinds of ignorance rather than knowledge and understanding. As we shall see shortly, *moral* vices are frequently appealed to in explaining extreme *behavior*, whereas *cognitive* vices are often referred to in explaining extreme *belief*.^7^

Second, some have suggested that vices are vices because of *bad motivations* (see Battaly 2014 for such a motivational component).^8^ Such motivational approaches are controversial, though. One might think, for instance, that someone who is narrow-minded may well be motivated by a desire for knowledge, it is just that she wrongly assesses the risks and thinks that something that conflicts with what she already believes is thereby likely to be false. Or one might think that someone who embraces David Icke’s reptilian thesis and other conspiracy theories out of credulity may do so from a psychiatric disorder. Perhaps, vice explanations and pathological explanations are perfectly compatible and in some cases go well together.^9^ Here, I will not take a stance on this and will not build motivational components into our understanding of cognitive vices.

Third, what is it to provide a *vice explanation* in the first place? What is it, for instance, to say that a person’s conspiracist belief is *explained by* his closed-mindedness? For reasons that I have spelled out elsewhere in more detail,^10^ I will understand it to mean this: In the case of a *full* explanation: that that person would not have held that belief if he had not been closed-minded, and in the case of a partial explanation: that that person held that belief partly because of his narrow-mindedness, even though other factors contributed to it as well.

Fourth, would it not be unduly pejorative to explain phenomena like conspiracism, fundamentalism, and extremism in terms of vices? As I have argued elsewhere, it *is* problematically pejorative – for instance, because it impedes fruitful use of definitions – to *define* phenomena like fundamentalism in negative terms, such as unwarranted affections or grievances, and vices. The issue under consideration is different, though: the purpose here is to *explain* extreme behavior and extreme belief. A full explanation of, say, violent extremism, may well have to appeal to various morally problematic character traits, like hatred and vengefulness, and some extreme beliefs can perhaps only be explained by appeal to cognitively problematic phenomena, like cognitive vices.

## Vice Explanations of Extreme Behavior and Extreme Belief

Vice explanations of conspiracy theorizing, fundamentalism, but particularly violent extremism are common in the literature. This is not to say that they are all right or solidly empirically based. The point is rather that they can be found frequently and that we, therefore, need to consider whether they can meet the situationist challenge.

It is not uncommon to explain fundamentalists’ or extremists’ behavior in terms of moral vices. Clark McCauley and Sophia Moskalenko explain Abu Musab al-Zarqawi’s radicalization towards brutal terrorism in Afghanistan partly in terms of his moral character traits like “aggressiveness” (McCauley and Moskalenko 2017, 78–82). This is clearly a *moral vice* explanation of al-Zarqawi’s heinous extremist deeds: to be aggressive is not to display aggression all the time, but to have a disposition to be aggressive in a wide range of circumstances, a disposition that was clearly triggered and manifested on numerous occasions in al-Zarqawi’s case. We also find moral vice explanations for fundamentalism. Munson (2008, 700), for instance, in explaining contemporary Islamic fundamentalism, appeals to the *hostility* that was already present among Muslims towards unbelievers and that was infused with new meaning when, due to processes of colonialization by Western empires, the distinctions between colonized and colonizer as well as oppressed and oppressor were added to that of believer and unbeliever. Hostility as well is best understood in terms of a disposition or character trait of the group: the idea in this quote is clearly not that Muslim fundamentalists continuously display hostile behavior, but that in relevant confrontation, they are hostile towards unbelievers.

But there is more than that. In explaining conspiracist, fundamentalist, and extremist *beliefs*, scholars even more often appeal to a wide variety of *cognitive* vices. Of course, if you ask a fundamentalist or conspiracist to explain *why* she believes a particular thing – say, that vaccination against Covid-19 is a way for the government to control the population – she will give you various reasons. She will refer to the testimony of witnesses, allegedly ‘scientific’ research on the harms of vaccination, and so on. And, of course, she may well hold these extreme beliefs *because* she takes these things to be good reasons to hold these beliefs. Yet, the idea of vice explanations is that in such cases, there is a *deeper* explanation: what truly explains her belief is, say, that she is gullible and prejudiced. This explanation is deeper, because it explains why she takes those reasons to be reasons to hold this belief about vaccination, why she discards evidence to the contrary, and why she does not consider further evidence.

Some evoke the vice of *prejudice* to define and explain fundamentalism. According to Douglas Lawrie, fundamentalists not only *have* biases and prejudices, but believe that such biases and prejudices are inevitable and justified (Lawrie 2008, 413). According to J.M. Vorster, religious fundamentalism’s ideologies “are known for their prejudice when faced with anything new or alien to their own strict ideas and morals. (…) The reactionary nature of religious fundamentalism is the root cause for its prejudice against otherness and its intolerance towards other ideas in its own midst.” (Vorster 2008, 51, 53).

Fundamentalism is sometimes characterized or explained in terms of *moral blindness* (e.g. Gupta and Kruthika 2003, 29), even though it is hardly ever explained what that amounts to or even why we should think so. I suggest that this notion is best understood as a cognitive vice: in the same way as visual blindness is a disposition to miss out on visual information, moral blindness is a disposition to miss out on moral truth (and to embrace moral falsehood): one is systematically unable to tell moral truths from moral falsehoods. Others speak of an “inability to enter the moral language game” (Sádaba 2003, 51).

Extremism is sometimes explained in terms of *black and white thinking*: “Extremists are often depicted as people who see the world in simplistic black- and-white terms rather than more nuanced shades of grey. This perspective is well illustrated in work linking extremism to individual deficiencies in cognitive complexity.” (Hopkins and Kahani-Hopkins 2009, 99) and with the “fundamentalist mindset” comes a tendency “to view the world in stark black and white terms, to simplify reality, to eliminate the gray zones, to perceive the self and others as part objects, and a gross failure to empathize with, or understand the inner lives of others—a failure to mentalize” (Reid Meloy 2018, 13).

Yet another vice is *stigmatization* or *demonization*. Here, the idea is that some extreme beliefs can be explained by the stigmatization or, to put it differently, demonization of the out-group. An example of this is what Sue Mahan and Pamala Griset say about extremist beliefs: “The term *right-wing religion,* as used in this article, refers to belief systems that incorporate some form of hatred or racism in their basic doctrines. There are four prominent forms of these theologies in America today: Christian Identity, Nordic Christianity or Odinism, free- wheeling fundamentalism, and Creatorism. These theologies are extremist religions based on the demonization of other racial, religious, or national groups.” (Mahan and Griset 2008, 192).

Particularly in explaining fundamentalism, scholars appeal to the cognitive vice of *dogmatism*: Fundamentalists “tend to be dogmatic. Scientific evidence that shows they are wrong would simply be ignored. They cannot conceive of anything that would lead them to change their belief in God.” (Altemeyer and Hunsberger 2005, 385), they have “a disposition towards dogmatism” (Pfürtner 1997, 107), and they are dogmatic and intolerant (Giedrojc 2010, 429). “Fundamentalism emphasizes moralism, dogmatism, and purism in the face of modern trends like consumerism, hedonism, and dissipationism.” (Abi-Hashem and Plante 2013, 242) “Another aspect of fundamentalists’ militant-combative attitude should be noted, namely the dogmatism that goes hand in hand with it but is distinguishable from it.” (Crawford 2014, 39) In fundamentalism, dogmatism replaces reflection (Ellis 2010, 60).

Finally, conspiracy theorizing is usually explained by various *cognitive* rather than *moral* vices. Prominent among them is *gullibility* and, closely related to it, motivated reasoning and confirmation bias. Evidence for this is, among other things, the fact that conspiracy thinkers are more prone to embrace paranormal beliefs, display more so-called ‘bullshit-receptivity’, and have more hyperactive agency detection (Van Prooijen 2019).

We should note that some authors clearly have cognitive vices like dogmatism in mind even if they do not use the vice terminology explicitly. An example of this are Naji Abi-Hashem and Thomas Plante when they say: “They [fundamentalists; RP] tend to lock themselves blindly to their doctrine or their past without sorting the complex matters, finding a middle ground, and modifying the stands. Their own values, norms, truths, practices, doctrines, and legacies represent their cherished tradition. Thus, they cling to these, rigidly unwilling to negotiate the relevancy of their beliefs and traditions to modern-day times.” (Abi-Hashem and Plante 2013, 240).

I conclude that vice explanations, both in terms of *moral* and in terms of *cognitive* vices, for various kinds of extreme behavior and extreme belief are common in the literature on conspiracism, fundamentalism, and extremism.

## The Situationist Challenge: Vices as Fictions

We have seen that cognitive vices figure widely in explanations of conspiracism, fundamentalism, and extremism—both actions and beliefs. Yet, there is a formidable challenge to all such explanations: situationism. The basic idea of situationism is that people are highly susceptible to various trivial and morally or epistemically irrelevant situational influences. People’s behavior and their beliefs are much better explained by appeal to such factors than by appeal to alleged moral or cognitive character traits. Among such irrelevant influences are mood depressors, ambient smells, ambient sounds, social distance cues, mood elevators, and the weather.^11^ Stronger versions of situationism say that there are no character traits as ordinarily conceived (Harman 1999, 2000), while more moderate versions say that there are no *robust* character traits (Doris 2002). Situationism in one form or another has been defended by Harman (1999, 2000, 2003), Doris (2002), Stephen Stich (Doris and Stich 2005), Alfano (2012, 2013), and Ross and Nisbett (2011). The evidence for situationism is a series of experiments, the most important ones of which are in chronological order:

1. Hartshorne and May (1928) tested students for whether they would cheat on exams. It turned out that there is no generic answer to this question: students would cheat on some tests, such as mathematics, but not on others, such as biology. This suggests that there is at most local consistency among the virtues and vices, things like *not cheating on tests in one area*, nothing across the board like honesty simpliciter.^12^
2. Isen and Levin (1972) performed two experiments in which they tested whether subjects are willing to help someone in need, say, a classmate or someone who accidentally dropped something. The best predictor turned out to be whether they were given a cookie in advance and whether they had found a dime prior to meeting the person in need. This suggests that behavior is not best explained in terms of stable character traits but in terms of often random situational factors.
3. Darley and Batson (1973) construed the seminal Good Samaritan experiment. Participants from Princeton Theological Seminary that were to deliver a talk on a good Samaritan related topic were, unbeknownst to them, brought into a scenario in which they encountered someone in need. It turns out that the best predictor for whether they would help them was not their view of religion, but whether or not they think they were running late. This might be taken to defeat the idea of robust character traits like benevolence, friendliness, altruism, and compassion. Again, behavior is best explained and predicted on the basic of external, situational factors.
4. People are less likely to help someone when there are loud noises in the background (Matthews and Cannon 1975) or bad smells (Baron 1997) in comparison with normal scenarios or scenarios with pleasant sounds and smells. People in positive moods are more likely to help than those in a bad or neutral mood (Weyant 1978). Again, external situational factors seem to matter more in explaining people’s behavior than alleged character traits.
5. All this would, of course, spell trouble for moral vice explanations for conspiracist, fundamentalist, and extremist *behavior*. However, vice explanations for extreme *beliefs* are not safe either because the idea that there are *cognitive* virtues and vices has also been questioned. According to Mark Alfano, “[m]any people do not possess creativity, flexibility and curiosity as such but inquire and reason creatively, flexibly and curiously when their moods have been elevated by such seemingly trivial and epistemically irrelevant situational influences as candy, success at anagrams, and comedy films.” (Alfano 2012, 239) Of course, there is further evidence to back up such claims. Whether people can solve a problem with four items depends on whether they are presented together (matches in a box) or separately (matches as one item, box as another) (Duncker 1945). Subjects perform better in the so-called remote associates test if they first get some candy, watch a comedy, or have their moods elevated in some other way (Isen et al. 1987). And so on. The suggestion is that people are not creative, original, or mentally flexible *simpliciter*, but, say, creative *when in a good mood or when positively affected*.

The general point of situationism should be clear by now: since various situational factors strongly influence people’s behavior and are better predictors than any alleged stable character traits, there is good reason to think that people have no robust moral or cognitive virtues or vices. Or, in Mark Alfano’s words:

The situationist critique (…) proceeds by pointing out that if people are unconsciously susceptible to such seemingly trivial and normatively irrelevant influences as their degree of hurry, receiving cookies, and finding dimes, one can only infer that they would also be swayed by major temptations. Situationist experiments suggest that most people do not even have flimsy virtues let alone robust ones. (Alfano 2013, 37)^13^

Experiments on moral virtues are mostly confined to honesty and willingness to help, whereas experiments on cognitive virtues are mostly confined to originality, creativity, and flexibility, but if the existence of these character traits is dubitable, then it seems so are the other virtues and vices.

Situationism should, of course, be understood as a critique of vice explanations for behavior and belief *in general*. As a wider skeptical objection, though, it also casts doubt on vice explanations for *extreme* behavior and *extreme* belief that, as we saw, are common in the literature on conspiracism, fundamentalism, and extremism. The alternative, suggested by situationist arguments, is, of course, to explain extreme behavior and extreme belief by appeal to situational factors—factors that have little to do with a person’s actual evidence and reasons.

And, in fact, such situational explanations abound in the literature. For instance, conspiracism has been explained in terms of poverty, marginalization, lack of control, low self-esteem, adverse childhood experiences, and unhappiness (Freeman and Bentall 2017), the extreme beliefs and extreme actions of terrorists have been explained by neurotic hostility with split personality (Post 1987, 308) and narcissism (Pearlstein 1991, 171). Of course, these explanations are not random in the sense that, say, smells and candy are. Yet, they also appeal to factors beyond the individual’s beliefs, reasons, arguments, narratives, and motivations. What the factors they appeal to (poverty, low self-esteem, unhappiness, split personality, and so on), have in common with the factors appealed to by situationists is that the subjects in question – fundamentalists, conspiracists, extremists – are not aware of them and would not appeal to them themselves in explaining or motivating their belief and behavior. Should we conclude, then, that situationism gives us good reason to prefer explanations of conspiracism, fundamentalism, and extremism in various social, economic, and political situational terms over vice explanations?

## Assessing Situationism

Here, I will present what I consider to be three important problems for situationist challenges to vice explanations. However, before I do so, let me voice three important preliminary worries about these studies (so, six worries or objections in total).

First, situationists use certain empirical evidence that seems to disqualify the notion of cognitive vice and simply leave aside empirical evidence that confirms the idea that cognitive vices shape our beliefs. It turns out, for instance, that the best predictor for belief in conspiracy theory is the so-called Conspiracy Mentality Questionnaire (CMQ) devised by Bruder et al. (2013). If anything, the questionnaire seems to track character traits (as Cassam 2016, 171 rightly points out). Therefore, if all we could do is simply consider the evidence, we should probably *suspend judgment* on whether some conspiracist, fundamentalist, and extremist beliefs and behavior can be explained by cognitive and moral vices.

Second, over the last few years, we have seen the enfolding of the so-called *replication crisis*: Large numbers of studies in various academic fields, including social psychology, turn out, upon attempts to replicate them, to lead to insufficiently similar results. Particularly important here are various so-called ‘many-labs’ collaborations meant to replicate studies about framing, certain kinds of priming, and embodied metaphor effects—these replication attempts were almost entirely unsuccessful (see Klein et al. 2014; Klein et al. 2018; Ebersole et al. 2016). Some have even withdrawn their earlier arguments for situationism on the basis of these failures to replicate (see, for instance, Alfano 2022). Others, though, might think that that is a little premature, because there is as yet no reason to think that these particular studies do not replicate. Since this would lead us into a complicated debate in the philosophy of science about how failures to replicate at a particular level should affect the trustworthiness of studies at other levels, I will leave the issue aside here and assume, merely for the sake of argument, that these studies would actually replicate.^14^

Third, as Quassim Cassam and others have pointed out (Cassam 2019, 45), situationism overlooks a critical difference between virtues and vices: virtues seem to require a considerable amount of consistency, whereas many vices do not. For example, one is an honest person only if one is honest most of the time. One cannot properly say that one is honest because one lies at most once a day or only if one is in a bad mood. One *can* have the vice of cruelty, though, as Abu Musab al-Zarqawi did, even if one is not consistently cruel, say, because one is only cruel when one is in a bad mood. We would not say someone is not cruel merely because he horrendously tortures his prisoners in only 40% of the cases in which he can do so. This means that the lack of consistency that the experiments that situationists appeal to draw our attention to, counts at most against the virtues but not against the vices.

Yet, one may worry that this first objection cannot all by itself deflate the situationist critique. A first consideration here is there are not only so-called *low-fidelity* but also *high-fidelity* vices: the former do not require consistency, but the latter do. Arguably, for instance, one is closed-minded only if one is so on most occasions. If one is regularly open-minded, it seems problematic to say that one is closed- or narrow-minded. I reply that it is quite possible, however, that some people are narrow-minded on nearly *all* relevant occasions, for instance, both when they are in a good mood and when they are in a bad mood. Not only have most situationist experiments been confined to virtues without considering the vices, what situationists have found so far is merely that many people do not *consistently behave well* across situations with a good and bad mood, not that there is not a considerable amount of people who *consistently behave badly* across situations with a good and bad mood. This first consideration, therefore, is not that worrying.

A second and more worrying point is that it has been argued that some extreme beliefs, such as belief in various conspiracy theories, are often formed not merely by the exercise cognitive vices but by the operation of cognitive vices *in combination with various cognitive virtues*. For instance, conspiracy theorizers form their beliefs not only from gullibility and undue skepticism, but from a desire for truth and intellectual perseverance—virtues they may possess to a higher degree than most people who simply do not care to delve into the evidence for, say, climate change or the safety of Covid-19 vaccinations. Such virtues include fundamentalists’ deeply caring about knowing the truth (Frykenberg 1997; Nipkow 2017) and, given that they often embrace a minority position (Baurmann et al. 2014), maybe even intellectual courage. To fully explain extreme beliefs, then, we sometimes also need to appeal to cognitive *virtues*. I will, therefore, go beyond this third reply to situationism and provide various further objections.

Fourth, what has been overlooked so far is that, as common-sense philosophers have argued in detail, common sense beliefs, concepts, and theories are often *vague* and *ambiguous*, precisely because they arose to be useful in daily life. This is not a deficiency, but actually a strength of common sense: it does not come with heavy metaphysical or epistemic baggage.^15^ Common sense, for instance, tells us that we have free will, but what exactly such free will amounts to and how it relates to determinism is not something on which common sense rules.^16^

When it comes to character traits, the vagueness of common sense is relevant on at least two points. On the one hand, common sense does not tell us how *domain-consistent* a vice is supposed to be. For instance, the claim in the literature on conspiracism is not that a conspiracist is *consistently* narrow-minded towards just anything. She may well be open-minded about sport, for instance. The claim *is* that a conspiracist is consistently narrow-minded across *some* domains. For instance, belief in one conspiracy theory makes it more likely that one will embrace further conspiracy theories. On the other hand, common sense is vague is that it does not tell us exactly *how much* explanatory work cognitive vices do. It does not tell us, for instance, that extreme beliefs can be explained *entirely* in terms of someone’s cognitive character traits; undoubtedly, various (sometimes irrelevant) situational factors also play a role. I submit that this is also how the literature on vice explanations for extreme beliefs ought to be understood: the claim is merely that cognitive vices *figure in* explanations of extreme beliefs, not that they fully explain these beliefs all by themselves.

Fifth, we should be careful *not* to conclude from situationist experiments that there are no virtues or vices or that there are no robust virtues and vices; only that they may be *rarer* than is sometimes thought (for a similar suggestion, see King 2014). For instance, many people (75–85%) would help someone in need, but only few (10–31%) if one or more impassive confederates are present. This is a result that has been systematically replicated, as meta-studies show (e.g., Latané and Nida 1981). If 10–31% of people would help someone in need even if one or more impassive confederates is present, then 10–31% of people *do* have the robust virtue of sympathy or willing to help: for them, the bystander effect does not obtain. This squares well with the previous point: situationism does not refute common sense vice explanations, but shows that they may be rarer than some people think. We should note, though, that this is *no* problem for explaining extreme beliefs, for *by definition* such beliefs are relatively rare—conspiracists, fundamentalists, and extremists are virtually always a minority (but there are exceptions), so it is perfectly plausible to appeal to vice explanations to explain these extreme beliefs.

Sixth, situationism seems to treat virtues and vices as an all-or-nothing matter, but it is more in line with common sense and character trait explanations as we find them in the literature to treat them as coming in *degrees*. It is compatible with the evidence that some people consistently almost never help, whether or not they are in a hurry, that others consistently help out when they are not in a hurry, and that a couple consistently help out even when they are in a hurry. The proper interpretation of this experimental result may well be that the first group is *not virtuous at all* in this regard at all, that the second category is *somewhat virtuous*, and that the third category *is highly and robustly virtuous*.

These objections, particularly the final three, jointly deflate the situationist challenge by sketching a more detailed and more realistic picture of vice explanations, namely by pointing to their domain-specificity, the fact that virtues and vices may just be rarer than we thought, and by taking into account that virtues and vices come in degrees.

## Fine-tuning Vice Explanations for Conspiracism, Fundamentalism, and Extremism

Even though I have argued that situationism does not *defeat* vice explanations, what we have seen so far suggests that situationism teaches us much about how we should phrase vice explanations for extreme behavior and extreme belief. In this section, therefore, I shall sketch how vice explanations of these phenomena should be refined in light of the situationist critique.

First, vice explanations should be *domain specific* and explore *how far the domain stretches*. In that regard, scholars should start to treat vices more like affections, which are, after all, not as dispositional as vices. It is standard, for instance in talking about extremists’ hatred and grievance, to be explicit as to the objects of these attitudes: exactly what is one grieved about and towards whom is such grievance displayed: colonization, mass-migration from Arabic countries, the West, oppression, the Iraqi war, being unemployed, alleged meddling in elections? In some cases, the objects of grievance can be even more specific, such as: mass-migration to the Netherlands from Morocco and Turkey from the sixties onwards (for right-wing extremism in the Netherlands). We should become similarly sensitive when it comes to traits like dogmatism, narrow-mindedness, and black-and-white thinking: in what realms – morality, politics, science, the pharmaceutical industry, global affairs, big tech industry, race, religion – and towards which issues or people – liberal ethics, or the government, and so on – are these character traits to be found?

By ensuring that our vice explanations are domain specific, we can achieve empirical adequacy: many extremists, for instance, do not display hatred towards just anything, but towards, say, a particular ethnic minority, such as Jews or Arabs. And many conspiracy thinkers are not credulous about just anything: they are not when it comes to soccer results on television, but they are when it comes to the government’s true purposes in adopting a controversial Covid-19 policy. By doing so, our vice explanations also gain in predictive power: to the extent that such (partial) explanations come with predictions, they do not predict, say, dogmatism or narrow-mindedness about just anything, but about, say, religious matters or the alleged behavior of those outside the group, the ‘unbelievers’. Such fine-tuning by specifying the domain helps us avoid vice explanations that are empirically inadequate and lack predictive power.

Here are two examples of what goes wrong when such vice explanations fail to be domain-specific. George Ellis writes:

I have come to understand the essential nature of fundamentalism as being *a partial truth proclaimed as the whole truth*. Only one viewpoint is allowed on any issue; all others are false. This dogmatism is combined with *an inability to relate understanding to context*, holding on to one viewpoint independent of its relevance to a particular situation. To admit that what is important varies with context would undermine the fundamentalist’s need to use the same single issue as dominant in every situation. (Ellis 2010, 59-60)

Two vices are mentioned here in the conceptualization and explanation of fundamentalism: dogmatism and the ability to relate understanding to context. The problem is that there is no reason to think that fundamentalists are dogmatic about just anything or that they always fail to relativize the importance of something to its context: many fundamentalists may well be open-minded and curious when it comes to technological issues, the natural sciences, hobbies of various kinds, and much more, and they may well relativize, say, understanding economic developments to various contextual issues, such as recent political changes. To fail to see this comes with the risk of stereotyping and misidentifying fundamentalists, which easily leads to, say, overlooking important phenomena in ethnographic research. Here is another example, one from an essay on Islamic fundamentalism in Pakistan:

The most brutal and dangerous manifestation of religious revivalism is ‘fanaticism’, which surfaces when those who stand for it are highly motivated and determined to die for the sake of religion. They are morally and reason-wise blind. The fidayeen or the suicide squads of various terrorist organizations is a case in point. (Gupta and Kruthika 2003, 30)

Gupta and Kruthika characterize Islamic fundamentalists or fanatics (they use these terms interchangeably) in terms of ‘moral blindness’ and ‘blindness reason-wise’. The problem is that many of them are clearly not morally or rationally blind: they may take good care of their children, treat their wives with love, live in harmony with nature, be respectful towards the elderly in their community, and so on, and they may excel at theological scholarship or be thoroughly familiar with the deliverances of the natural sciences. One may wonder whether ‘blind’ is an apt term at all for the vice they display. Perhaps a term like ‘moral blindness’ is better reserved for, say, psychopaths who enjoy killing and seem to utterly lack a moral sense. When it comes to certain kinds of fundamentalists, it rather seems that they have become morally insensitive specifically regarding the rights or human dignity of Westerners, those of other faiths, homosexuals, and so on—if at all: maybe they are still sensitive but simply suppress such feelings or explain them away.

Sometimes, authors indicate that an extreme actor or extreme believer does not have a vice *simpliciter*, but that it is restricted to such a domain. It is not always clear exactly what that domain, according to the author, amounts to. Quassim Cassam has been a leading figure in (rightly) putting vice explanations for extreme belief on the agenda. He introduces a fictional character Oliver who embraces conspiracy theories about 9/11, as well as other conspiracy theories. He points out that an important explanation could be one in terms of cognitive vices:

Suddenly it all begins to make sense, but only because the focus has shifted from Oliver’s *reasons* to his *character*. You can now see his views about 9/11 in the context of his intellectual conduct generally, and this opens up the possibility of a different and deeper explanation of his belief than the one he gives: he thinks that 9/11 was an inside job because he is gullible in a certain way. He has what social psychologists call a ‘conspiracy mentality’. (Cassam 2015)

Cassam is clearly fully aware that conspiracy theory believers need not be gullible across the board: they are gullible in a particular domain, gullible in a way that displays a conspiracy mentality. This, of course, raises questions about exactly what the domain is and what such a conspiracy mentality amounts to.

Some scholars who study fundamentalism, conspiracism, and extremism pay explicit attention to the domain-specificity of vices that serve as explanations. Altemeyer and Hunsberger, for instance, do not categorically claim that fundamentalists are biased, but that they “tend to be more *racially* prejudiced than most people are, if by small amounts” (Altemeyer and Hunsberger 2005, 385; italics mine). Jan-Willem van Prooijen and others in studying populism and conspiracy theorizing speak not of the vice of ‘credulity’ in general but specifically of credulity towards “obscure and politically neutral news items (regardless of whether they were broadcasted by mainstream or alternative news sources), receptivity to bullshit statements, and supernatural beliefs” (Van Prooijen et al. 2022) And Naji Abi-Hashem and Thomas Plante are even more explicit when they say:

Most fundamentalists are not skewed in all areas of life and intellect. Rather they are passionate and inflexible in certain spheres only (and that is true for political affiliations, social activists, secular lifestyle defenders, etc.). When challenged, they tend to over-react with apprehension, intensity, and resentment. Interestingly, the majority of them remain quite pleasant, reasonable, and functional in other areas of life and public domains. However, when hot topics are brought up and discussed, their core beliefs and sensitivities become stirred up and activated, so they react strongly in obsessive, defensive, and judgmental ways.^17^

Second, vice explanations should be fine-tuned by making explicit whether a *low- or high-fidelity vice* is involved and, therefore, *how much consistency* the vice in question requires. Take the cognitive vice of gullibility. Clearly, it is low-fidelity: if one believes the most extravagant claims on the basis of virtually no evidence whatsoever in only 30% of the relevant cases, one is still gullible. If a conspiracist and fundamentalist believes that *The Protocols of the Elders of Zion* shows that a couple of rich Jewish conspire together and seek world domination merely because his friend told him so, if he believes that a literal and historical interpretation of Genesis 1–3 has been dominant throughout most of the church’s history but merely because his pastor told him so, and if he holds a couple of similar beliefs on similar bases, he is gullible, even if the vast majority of his beliefs are similar to yours and mine in content and basis. Other vices are clearly high-fidelity. If someone in Bin Laden’s following agreed with him on some occasions, but disagreed with him on other occasions, saying that Bin Laden was radically mistaken in his interpretation of the Qur’an and that his actions were immoral all-things considered, we would not say that he suffered from the vice of conformity or the vice of intellectual cowardice. This is true even if that person adopted the views of Bin Laden on *some* occasions just because they were Bin Laden’s or out of fear. Conformity and cowardice, then, are high-fidelity vices.

Fine-tuning of vice explanations by paying attention to the low-fidelity/high-fidelity distinction will help us integrate theoretical (including philosophical) work on vices and empirical work on vice explanations: we thereby avoid appealing to vices where theorists deny the presence of that character trait. It will also help us make sense of the fact that some vice explanations have high predictive power, because they concern high-fidelity vices that require high frequency and relative consistency in a particular domain, whereas other vice explanations do not, because they concern low-fidelity vices that do not require high frequency or relative consistency in a particular domain.

Third, vice explanations should be *sensitive to relevant situational factors* and elucidate how much in an explanation is due to vice factors, how much to situational factors, and how much to yet other factors. For instance, in exploring the relation between the exemplification of cognitive vices – or, as they call them, epistemic vices – on the one hand and conspiracy theorizing on the other, the authors of the Epistemic Vice Scale write:

The Epistemic Vice Scale is internally consistent; has good convergent, divergent, and discriminant validity; and is strongly associated with the endorsement of misinformation and conspiracy theories. Epistemic vice explains additional variance in the endorsement of misinformation and conspiracy theories over and above demo- graphic and related psychological concepts and shows medium to large effect sizes across outcome measures.^18^

Here, they explicitly acknowledge that epistemic vices should figure in an explanation of conspiracy theorizing, but that demographic and other, related psychological concepts do so as well. Of course, it would have been even better if they had also acknowledged the potential role of factors beyond demographics, vice, and psychological properties, determinants like political situation, cultural factors, socio-economic status, and religiosity, but we can easily add that ourselves.^19^

What we gain by specifying the role or relative importance of vices in explaining extreme belief and behavior in comparison with other factors, such as situational factors, is that we do not ask or expect more from vice explanations than what they should be meant to do: provide a partial explanation which only becomes full when we appeal to further personal and situational explanatory factors. We avoid wrongly criticizing vice explanations for not fully explaining why specific individuals or groups embrace extreme beliefs, such as conspiratorial beliefs, and perform extreme actions, such as violent insurrectionist attacks.

## Objections and Replies

First, can these cognitive vices that are meant to explain conspiracism, fundamentalism, and extremism be *quantified* and *measured*? If not, how could they figure in explanations for extreme beliefs? My reply is twofold. First, many phenomena can figure in scientific explanations even if there is not (yet) a way to quantify and measure them. In-depth interviews and much of ethnography, for instance, do not require operationalizing, quantifying, or measuring, as, for instance, the literature on motivations for terrorism (De Gaaf 2021) and conspiracy theories (Harambam 2020) show. Second, cognitive vices *can* be quantified and measured, as the conspiracy and fundamentalism scales show. Here, for instance, is how Altemeyer measures the vice of dogmatism:

Dogmatism, defined as relatively unchangeable, unjustified certainty, is measured by a 20-item DOG Scale containing such statements as “The things I believe in are so completely true, I could never doubt them,” “There are no discoveries or facts that could possibly make me change my mind about the things that matter most in life,” and “I am so sure I am right about the important things in life, there is no evidence that could convince me otherwise” (Altemeyer and Hunsberger 2005. (…) religious fundamentalism has correlated .57–.78 with DOG scores in studies thus far, and as with zealotry, most of the highly dogmatic people one finds in a sample are religious fundamentalists. (Altemeyer and Hunsberger 2005, 382)

As we saw above, others have recently developed and validated what they call an Epistemic Vice Scale (Meyer et al. 2021), which includes the vices of closed-mindedness, sloppiness, obstinacy, apathy, and diffidence. In fact, they also found that they correlate with conspiracy thinking.

Second, one may object that vice explanations often cannot be used to accurately *predict* conspiracism, fundamentalism, and extremism. I agree. There is, however, an entirely plausible explanation for that. As numerous authors have argued, explanations of phenomena like fundamentalism are multi-layered. Almond et al. (2003), for instance, have argued that any viable explanation of fundamentalism will comprise three different kinds of factors: structural factors, contingency factors, and choice factors. Examples of structural factors are conflicts with other religions, war, economic crises, population movements, and cultural secularization. Examples of contingent chance factors are natural disasters and the natural death of a leader. And examples of choice factors are the decision to found a sect, the decision to commit a terrorist attack, and specific styles of leadership. Cognitive vices would presumably fall in the third and final category: even though one may not choose one’s vices, one can work on them and one can be responsible for them. If this is true for *explanations* of fundamentalism, then it is true *a fortiori* for *predictions* of fundamentalism: vices are only *elements in parts of* the explanation, vice explanations are not meant to provide a full explanation of extreme beliefs all by themselves.

Third, one may worry that people’s specific cognitive vices need to be explained at least partly in terms of their beliefs—in fact, sometimes in terms of their *extreme* beliefs. Fundamentalists and extremists may be narrow-minded when it comes to the moral status of out-groups precisely because they hold the belief that everyone outside the in-group lives a deeply immoral life. Or they can be unduly skeptical towards anything the government does precisely because they hold the view, propagated by David Icke, that the world’s governments are just puppets of reptilian shape-shifting aliens. If this is right, vice explanations for extreme beliefs face a *vicious regress*: extreme beliefs are explained by cognitive vices, those cognitive vices are explained by extreme beliefs, and so on.

This would indeed be a problem if the aim was to fully explain all extreme behavior and belief by appeal to vices. Fortunately, though, the picture is much more complicated. Often, vices, especially cognitive vices, and beliefs mutually influence one another (affections, desires, and further influences also interact with these) until at some point a rather stable cognitive character with various vices has come about, vices that can then provide at least a partial explanation of why new extreme beliefs are accepted (coherence with other extreme beliefs will then be a further part of the explanation) and already present extreme beliefs maintained.

Fourth and finally, one might wonder whether there are not further explanations that are in some sense *deeper* than vice explanations. If one wonders, for instance, why so many people believe in QAnon conspiracy theories in the United States in 2023,^20^ one could refer to the dogmatism, narrow-mindedness, skepticism, prejudice, gullibility, and further cognitive vices that these conspiracy theorists seem to exemplify. And that would be correct in many cases. But a deeper explanation of these extreme beliefs would appeal to political factors, such as the rise of Trumpism and the turn of the Republican party towards protectionism, socio-political factors like increasing polarization, and international-political developments like the spread of Covid-19 and the attempts of numerous public institutions to counter global warming.

I reply that what this worry really shows is that we should carefully distinguish between *different kinds of explananda*, as has also been pointed out by Cassam (2019, 48–49). Note that in the above example the explanandum is *There being many people in the United States in 2023 that embrace QAnon conspiracy theories*. Here, a vice explanation will not do: even if all these beliefs are somehow the product of cognitive vices, the question is still why so many people nowadays in the United States – in comparison with some other countries in the world and some other time periods – embrace conspiracist beliefs like those propagated by QAnon. Numerous other people in other countries and other time periods also suffered from cognitive vices, so an explanation in terms of cognitive vices will not do. An explanation in terms of socio-political-cultural factors may well do. Compare this with the question of why, say, Alex Jones is embracing various extreme conspiracy theories, like the theory that a New World Order is coming, a “demonic high-tech tyranny”, and that it is formed by Satanist elites that manufacture various economic and health crises. Here, socio-political-cultural factors are less relevant, because many other contemporary Americans *reject* such conspiracy theories. Specific cognitive vices that Alex Jones may have could figure well in an explanation of why he holds these extreme beliefs.

## Conclusions

We have considered the situationist challenge to vice explanations for extreme behavior and extreme belief, since such explanations are common in the literature on conspiracism, fundamentalism, and extremism. Some take a large number of experiments to count against vice explanations. Upon a closer look at the evidence leveled by these experiments, though, it turned out that vice explanations should not be discarded altogether, but merely fine-tuned. The main reason for this is threefold: our beliefs about vices are somewhat ambiguous and can easily be revised, the evidence does not suggest that there are no vices but at most that they might be rarer than often thought, and the reasoning from these experiments fails to take sufficiently into consideration that both virtues and vices come in degrees. What the experiments suggest is that vice explanations should be fine-tuned: Vices are often domain-specific rather than general, some cognitive vices are low-fidelity, whereas others are high-fidelity, and vice explanations provide at most *partial* explanations: a full explanation of conspiracist, fundamentalist, and extremist behavior and belief should take numerous situational factors into account.

## Footnotes

1 For a more detailed overview of what are probably the metaphysical constituents of fundamentalism and related phenomena, see Kindermann, Peels, Liefbroer, Schoonmade, (n.d.) unpublished manuscript.

2 See Peels, Tietjen, Berger, Horgan (2024).

3 Reality is complex here, though: other beliefs that are clearly extreme in some sense, such as delusional beliefs about alien abduction, have *not* fallen under the purview of vice epistemology in the literature, even though they seem equally extreme and in fact equally irrational. It is an interesting question why that is. One explanation is that one might think that such monothematic delusional beliefs are best explained by anomalous experience in combination with normal cognitive variation across a general population (see Sullivan-Bissett 2020; Noordhof and Sullivan-Bissett forthcoming). Others, though, appeal to performance error, bias, or cognitive deficit, so this would leave room for explanations in terms of cognitive vices.

4 They are also called *epistemic* or *intellectual* vices.

5 Similarly, Cassam defines cognitive vices as traits that “impede effective and responsible inquiry” (Cassam 2016, 159), as traits and thinking styles that “get in the way of the gaining, keeping or sharing of knowledge” (2022) and yet elsewhere he defines ‘cognitive vice’ as a personal quality “that gets in the way of knowledge or understanding” (2021).

6 For lists along these lines, see Cassam (2019), Zagzebski (1996, 152). Cognitive *virtues*, then, are character traits that are knowledge-conducive, traits like attentiveness, benevolence, carefulness, creativity, curiosity, discernment, fairmindedness, flexibility, honesty, humility, integrity, objectivity, open-mindedness, originality, parsimony, perseverance, studiousness, thoroughness, understanding, vigor, warranty, and wisdom. Another cognitive vice is *epistemic insouciance* (not caring about the truth), but it seems that vice will not figure prominently in explanations of extreme beliefs because such beliefs are formed precisely because one cares about the truth (otherwise one could just suspend judgment or not even bother to consider the issue).

7 What sort of a thing is a cognitive vice? There are at least two different conceptualizations of virtue and vice in the literature. On a narrow conception, they are character traits. On a broader conception, virtues are excellences and vices defects. Thus, among the cognitive virtues are also good eyesight and capacious memory, while among the cognitive vices are things like forming beliefs by guesswork, wishful thinking, and ignoring contrary evidence are vices. For different conceptions of virtues and vices, see Axtell (2000), Battaly (2015) and Baehr (2011). This article zooms in on virtues and vices *as character traits*; these, after all, are the most controversial ones. If there is good reason to think that there *are* cognitive character traits that explain some of our behavior and beliefs, then this will be true *a fortiori* for the other cognitive virtues and vices.

8 At the background here is the disagreement between *reliabilists*, who argue that cognitive virtues and vices do not require specific motivations, and *responsibilists*, who claim that cognitive virtues come with a desire for truth, knowledge, and understanding, while the cognitive vices come with no such desire or even a desire for their opposites.

9 To what extent pathological explanations of conspiracy theory belief are viable and whether in some cases conspiracy theory belief ought to be understood as a delusion, is studied in detail by various other articles in this special issue.

10 See Rik Peels, "What Is It to Explain Extremism?", unpublished manuscript.

11 Some have categorized these influences into (i) bad reasons, such as the unresponsive bystander effect, (ii) situational non-reasons, such as ambient smells and sounds, and (iii) non-moral individual differences, such as culture and gender (Alfano 2013, 35–53).

12 Similar results were found by Ariely (2008) when he conducted experiments with Harvard and MIT students who could decide to cheat or not to cheat on exams. Among other things, if they were asked to write down as many of the ten commandments as they could remember, they would cheat significantly less.

13 So, why do people mistakenly ascribe virtues and vices? Multiple and mutually compatible answers are given by situationists (e.g., Alfano 2013, 54–61): *attribution error*, that is, the tendency to attribute observable behavior to internal dispositional factors rather than external ones; *false consensus effect*, that is, the false assumption that one’s own belief is representative of that of others; *confirmation bias*, *availability bias*, *selection bias*, and so on.

14 An additional worry with situationist experiments has to do with research integrity: one might think that too many studies published prior to 2012 suffer from questionable research practices, such as *p*-hacking, as a reviewer for this journal suggested. This may be right, but would lead us into different territory. Therefore, I will instead focus on four criticisms here that, for the sake of argument, take the findings of these experiments for granted.

15 E.g. Rescher 2005.

16 Thus also Haselager 2020.

17 Abi-Hashem and Plante 2013, 241.

18 See Meyer et al. 2021.

19 By way of example, for the latter, see Dawson 2021a, b.

20 Some 17%, according to Bloom and Moskalenko (2021).
